# Effect of isolated and combined ingestion of caffeine and citrulline malate on resistance exercise and jumping performance: a randomized double-blind placebo-controlled crossover study

**DOI:** 10.1007/s00394-023-03212-x

**Published:** 2023-07-14

**Authors:** Markus Estifanos Haugen, Fredrik Tonstad Vårvik, Jozo Grgic, Henrik Studsrud, Espen Austheim, Erik Mathias Zimmermann, Hallvard Nygaard Falch, Stian Larsen, Roland van den Tillaar, Thomas Bjørnsen

**Affiliations:** 1 Department of Sport Sciences and Physical Education, Nord University, Levanger, Norway; 2 Department of Sport Science and Physical Education, Faculty of Health and Sport Sciences, University of Agder, Kristiansand, Norway; 3 Norwegian Olympic and Paralympic Committee and Confederation of Sports, Oslo, Norway; 4 Institute for Health and Sport, Victoria University, Melbourne, Australia

**Keywords:** Nutritional supplements, Sports nutrition, Resistance training, Hypertrophy, Bench press, Squat, Force, Power

## Abstract

**Purpose:**

The aim of this study was to explore the isolated and combined effects of caffeine and citrulline malate (CitMal) on jumping performance, muscular strength, muscular endurance, and pain perception in resistance-trained participants.

**Methods:**

Using a randomized and double-blind study design, 35 resistance-trained males (*n* = 18) and females (*n* = 17) completed four testing sessions following the ingestion of isolated caffeine (5 mg/kg), isolated CitMal (12 g), combined doses of caffeine and CitMal, and placebo. Supplements were ingested 60 min before performing a countermovement jump (CMJ) test (outcomes included jump height, rate of force development, peak force, and peak power), one-repetition maximum (1RM) squat and bench press, and repetitions to muscular failure in the squat and bench press with 60% of 1RM. Pain perception was evaluated following the repetitions to failure tests. The study was registered at ISRCTN (registration number: ISRCTN11694009).

**Results:**

Compared to the placebo condition, isolated caffeine ingestion and co-ingestion of caffeine and CitMal significantly enhanced strength in 1RM bench press (Cohen’s *d*: 0.05–0.06; 2.5–2.7%), muscular endurance in the squat (*d*: 0.46–0.58; 18.6–18.7%) and bench press (*d*: 0.48–0.64; 9.3–9.5%). However, there was no significant difference between isolated caffeine ingestion and caffeine co-ingested with CitMal, and isolated CitMal supplementation did not have an ergogenic effect in any outcome. No main effect of condition was found in the analysis for CMJ-derived variables, 1RM squat and pain perception.

**Conclusion:**

Caffeine ingestion appears to be ergogenic for muscular strength and muscular endurance, while adding CitMal does not seem to further enhance these effects.

**Supplementary Information:**

The online version contains supplementary material available at 10.1007/s00394-023-03212-x.

## Introduction

Caffeine is a highly popular ergogenic supplement used among athletes and non-athletes alike [1]. Previous research established that caffeine ingestion, usually in doses from 3 to 6 mg per kg of body mass, may provide an ergogenic effect on various exercise outcomes, such as aerobic and muscular endurance, maximal strength, power, and jumping performance [2–4]. This ergogenic effect is commonly coupled with a reduction in subjective rating of perceived exertion (RPE) during exercise [5]. From a mechanistic standpoint, it is important to emphasize that caffeine has a similar molecular structure to adenosine. Therefore, after ingestion, caffeine acts as an adenosine receptor antagonist (i.e., caffeine binds to adenosine receptors) [6]. By binding to these receptors, caffeine may reduce fatigue sensations, as well as increase cognitive and physical readiness, which can contribute to improvements in exercise performance [7]. The effect of caffeine on adenosine receptors is the most likely explanation for its ergogenic effect. However, it has also been suggested that other mechanisms, such as the direct effects of caffeine on skeletal muscle, may contribute to performance improvements following caffeine ingestion, even though these findings are mostly observed using animal models [8].

Another supplement that has gained popularity in recent years is citrulline malate (CitMal), which represents a combination of L-citrulline and malic acid. L-citrulline is a non-essential amino acid primarily found in foods such as watermelon and cucumber [9]. L-citrulline is a precursor to L-arginine [10], as L-citrulline is transported to the kidneys, where it can be directly converted to L-arginine [11]. L-arginine increases nitric oxide production [12]. One of the functions of nitric oxide is that it induces vasodilatation (dilatation of the blood vessels). Vasodilatation may affect exercise performance through: (a) reduced blood pressure; (b) increased blood flow that may increase nutrient and oxygen delivery to the working muscle; and (c) increased clearance of metabolic by-products [12]. This could potentially reduce the energetic cost of ATP in a muscle contraction, enhance force production, and improve calcium handling and mitochondrial efficiency [13]. CitMal may also influence ammonia clearance and delay exercise-induced fatigue resulting with performance improvements [14]. Indeed, an animal-model study reported that citrulline consumption reduced ammonia accumulation and increased endurance performance [15]. Due to these physiological effects, supplementing with CitMal can induce a small acute ergogenic effect on strength and power performance [16], muscular endurance [17], and reduce post-exercise RPE and muscle soreness [18]. Most commonly, doses ranging from 6 to 12 g of CitMal are used to attain acute performance benefits [17]. These doses are generally safe and well-tolerated in humans. For example, one study used 2, 5, 10 and 15 g of citrulline and reported that none of the participants experienced any side effects, regardless of the consumed dose [19].

Historically, research has mainly explored the effects of a given supplement when provided in isolation. This approach is adopted from a methodological standpoint as it allows for the controlled intake of other substances that may enhance exercise performance [20]. While such an approach is certainly appropriate from a research standpoint, it does not allow us insights into the combined effects of multiple supplements. Exploring the combined effects of different supplements is important given that athletes commonly use several supplements to maximize performance gains and different ingredients are mixed in pre-made or self-made pre-workout supplements [20]. To the authors’ knowledge, no previous study has investigated the combined effects of caffeine and CitMal on exercise performance. Therefore, the aim of this study was to explore the isolated and combined effects of caffeine and CitMal on jumping performance, muscular strength, muscular endurance, and pain perception. We hypothesized that both caffeine and CitMal would be ergogenic and that their combined ingestion would produce additive effects on exercise performance.

## Methods

### Study design

**Fig. 1:**
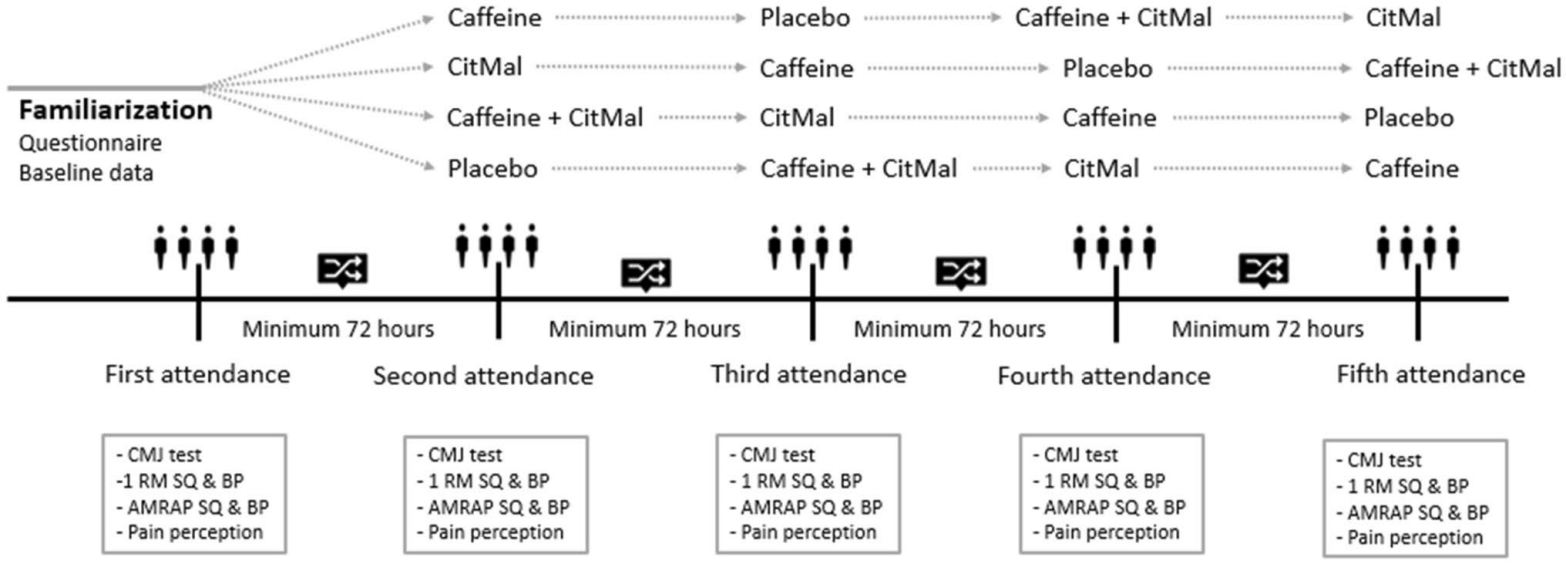
Overview of the study design. CMJ countermovement jump, RM repetitions maximum, SQ squat, BP bench press, AMRAP as many repetitions as possible, PP pain perception

**Fig. 2:**
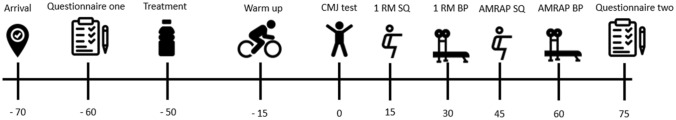
Timeline of test day. Questionnaire one was only given on familiarization (personal data, weight, food log, caffeine log, resistance training experience). Questionnaire two included evaluation of the effectiveness of the blinding. CMJ countermovement jump, RM repetition maximum, SQ squat, BP bench press, AMRAP as many repetitions as possible

A randomized, double-blind, placebo-controlled, crossover trial (Fig. 1) was used to investigate the isolated and combined effects of caffeine and CitMal on countermovement jump (CMJ) performance, maximal strength, muscular endurance, and pain perception. Sample size was calculated with a power analysis (G*Power V 3.1.9.6) and it indicated that 36 participants were required. The calculation was based on a small effect size (Cohen’s d = 0.20) for maximal strength an alpha level of 0.05, statistical power of 80%, correlation of r = 0.5, one group with 4 measurements, for repeated measures, within subject analysis of variance (ANOVA). In the first session, the participants were familiarized with the testing protocol, which was then repeated for all the experimental sessions. The experimental sessions involved four testing trials where the participants received either placebo (zero-calorie drink), 5 mg/kg caffeine, 12 g of CitMal, or the respective dosages of caffeine and CitMal combined, mixed in 500 mL non-caloric cordial. In all four conditions, supplementation was provided 60 min before the start of the testing protocol. All testing trials were separated by a minimum of 72 h and a maximum of seven days to ensure treatment washout and sufficient recovery. The participants were instructed to refrain from caffeine, alcohol, and training 24 h prior to every testing session. During the course of the study, the participants were instructed not to consume any supplements. All participants recorded a 24 h diet log the day prior to testing and a weekly caffeine log. The participants were instructed to replicate the food log before every trial to reduce variation in energy intake and hydration level. The order of the trials was counterbalanced and randomized using Microsoft Excel (Version 2205, 64-bit) (Fig. 2).

### Participants

Eligibility criteria for participation included: (a) being 18–45 years old; (b) possessing a minimum of 12 months of resistance training experience; (c) currently performing resistance training at least two times per week; and (d) able to perform the barbell back squat and bench press with 120% and 100% of body mass for males and 100% and 70% of body mass for females, respectively [21]. Participants were excluded if they were smokers, pregnant, or lactating. A total of 40 healthy resistance-trained males (n = 20) and females (n = 20) with no known medical condition, injuries, or any other health limitation were initially recruited. A total of 35 participants, 18 males and 17 females (age: 23.0 ± 3.2 years, 174.5 ± 9.9 cm, 76.9 ± 14.7 kg), completed all four trials and were included in the analysis (Table 1). Four participants dropped out due to injury sustained outside the experiment, and one was lost to follow-up (i.e., did not attend all testing sessions due to unspecified reasons) (Supplementary file 1). The study was performed in accordance with the Helsinki Declaration and approved by the Norwegian Center for Research Data (NSD) (project nr: 445723) and by the local ethics committee at the University of Agder (Kristiansand, Norway). All participants signed a written consent.

**Table 1:** Participant characteristics for those who completed the study

	Men (n = 18)	Women (n = 17)	All participants (n = 35)	
	Mean ± SD	Mean ± SD	Mean ± SD	Range
Age (years)	23.8 ± 3.4	22.1 ± 2.9	23 ± 3.2	18–30
Height (cm)	182.0 ± 7.3**	166.1 ± 3.1	174.5 ± 9.9	160–200
Body mass (kg)	87.2 ± 12.5**	65.5 ± 5.6	76.9 ± 14.7	58.8–118.8
Fat-free mass (kg)	67.4 ± 6.1**	46.3 ± 3.6	57.2 ± 11.8	39.8–80.3
Fat mass (%)	18.2 ± 6.6*	25.1 ± 6.0	21.9 ± 7.1	9.9–35.1
Resistance exercise experience (years)	4.0 ± 2.2	4.7 ± 2.3	4.3 ± 2.3	1.0–10
Squat experience (years)	2.7 ± 2.2	3.1 ± 1.6	2.9 ± 1.9	1.0–9.5
Bench press experience (years)	3.0 ± 2.2	3.1 ± 1.8	3.1 ± 2.0	0.5–9.5
Resistance training frequency (sessions /week)	4.6 ± 1.5	4.6 ± 1.5	4.6 ± 1.5	1–7
Energy (kcal)	2616 ± 522**	1787 ± 365	2224 ± 614	1138–3858
Protein (gram/day)	142 ± 41	110 ± 27	127 ± 38	70–210
Carbohydrate (gram/day)	314 ± 75**	199 ± 60	260 ± 90	83–462
Fat (gram/day)	88 ± 28*	61 ± 22	75 ± 29	22–165
Caffeine (mg/day)	332 ± 126*	206 ± 124	273 ± 139	25–487
Caffeine (mg/ kg body mass/day)	3.8 ± 1.4	3.2 ± 2.0	3.5 ± 1.7	0.4–7.5
Numbers of days with caffeine per week	6.4 ± 1.3	5.0 ± 1.9	5.8 ± 1.8	1.5–7

*1RM* 1 repetition maximum, *MG/DAY* mg per kg body mass per day, *GR/DAY* gram per day, *KG/body mass* kilogram per kg body mass, *RE* resistance exercise, *Numbers of days w caffeine* numbers of days with caffeine consumption on the weekly log

*Indicates a significant difference between men and women on a p < 0.05 level

**Indicates a significant difference between men and women on a p < 0.01 level. Differences between the groups were examined using an independent t test

### Supplementation

All supplement conditions had a similar color and taste as they were mixed in 500 mL of Fun Light zero-calorie sweetened water (200 mL water and 300 mL non-caloric Fun light sweetener; Fun Light^©^*, Stabburet, Nordre Follo, Norway*), and consisted of: (1) 5 mg/kg of caffeine as an anhydrous powder (Caffeine, ReagentPlus, Sigma-Aldrich); (2) 12 g of CitMal powder with a ratio between L-citrulline and malate of 2:1 (Citrulline Malate, Trade Ingredients); (3) the same dosages of caffeine (5 mg/kg) and CitMal (12 g) combined; or (4) placebo (water and Fun Light sweetener). Participants were provided the drink in bottles 60 min prior to testing and were required to consume the whole drink within one minute to ensure that they reached peak or close to peak plasma levels of caffeine and CitMal when the tests were initiated [19, 22]. Bottles with supplements were shaken between every sip. An independent researcher who did not participate in other measurements or analysis randomized the treatment order, mixed, and administered the treatments. This researcher unblinded the conditions only after the data collection for all participants was completed. After every testing session, participants were asked which supplement they thought they had received. A standard question was given to all participants at the end of the testing sessions: “Which supplement do you think you received?” Answers included: placebo; only caffeine; both caffeine and CitMal; only CitMal.

### Adverse events

The incidence and severity of adverse events were tracked during the testing sessions and throughout the remainder of the testing day. Specifically, during the testing sessions, the participants were encouraged to provide details about any adverse events (e.g., nausea). After the testing was completed, the participants were also required to record any additional adverse events (e.g., insomnia, irregular heartbeat, etc.). If an adverse event occurred, the participants were required to note its description, the likelihood of its association with the intervention (not related, unlikely, possibly, probably, definite), severity (life-threatening, required hospitalization, resulted in persistent disability, or non-serious), and its intensity (mild, moderate, severe, life-threatening) [23].

### Measurements

#### Countermovement jump

Each testing session started with the CMJ test on a force plate (Muscle lab, Ergotest Technology AS, Porsgrunn, Norway), which was used to evaluate jump height (cm), maximal power (W), rate of force development (kN/s), and peak force (N/kg). The CMJ was performed with feet shoulder-width apart and hands on the hips during the whole jump. From a standing position, participants were required to squat to a self-selected depth and then perform a maximal vertical jump. The feet had to be straight during the flight time. Jump height was calculated with impulse (Muscle lab, Ergotest Technology AS, Porsgrunn, Norway). As a warm-up, the participants performed three submaximal CMJ trials with approximately 50%, 75%, and 90% intensity (45 s of rest between attempts). Following the warm-up, three CMJ attempts were performed, and the participants rested 15 s between each attempt. Highest values for each of the analyzed variables were used for statistical analysis.

#### 1RM squat and bench press

Participants completed a 1RM test for both the squat and the bench press. In both exercises, a five sets warm-up protocol was utilized. In the first set, the participants completed several repetitions only with the barbell. Then, they completed 8, 6, 3, and 2 repetitions with loads amounting to 40%, 60%, 70%, and 80% of their estimated 1RM, respectively [24]. 1RM attempts started following the completion of the warm-up sets. After every successful 1RM attempt, the weight was increased by 0.25 kg to 5 kg (subjectively evaluated) until a final 1RM was reached. Four minutes of rest were provided between 1RM attempts. Equipment used for the testing included: a half rack (half rack easy 2.0, ata Group AS, Asker, Norway), a calibrated (± 10 g) 20 kg barbell (ata Powerbar stainless steel 29 mm, ata Group AS, Asker, Norway), and calibrated (± 10 g) plates from 0.25 to 50 kg (ata Powerlifting Steel Plate, ata Group AS, Asker, Norway). For the squat, the participants were required to reach the depth requirement set by the International Powerlifting Federation [25], which requires that the top surface at the hip joint should be below the knees. A test leader visually inspected the depth together with a rubber band that the participants could use as external feedback. Safety pins and two experienced spotters were used in the 1RM attempts to ensure safety. In the bench press, the elbows had to be fully extended at the completion of the lift for a 1RM to be approved. A pause on the chest at the bottom of the lift was not mandatory (self-selected by the participants and standardized for all testing sessions). However, the shoes had to be in touch with the floor and the gluteal region and upper back in touch with the bench throughout the lift. Stance width in the squat and grip width in the bench press was recorded in the familiarization session, and the same width was used in all experimental sessions. Equipment such as lifting belts, shoes, wrist wraps, knee sleeves and chalk were allowed, but participants had to use the self-selected equipment at all trials to ensure standardization.

#### Repetitions to failure and pain perception

Muscular endurance was evaluated by having the participants complete one set of repetitions to muscular failure in the squat and bench press using 60% of 1RM (from 1RM measured at each trial). During the set, the number of completed repetitions was counted out loud by the test leader. No breaks were allowed between repetitions. Technical requirements were the same as in the 1RM tests. Muscular failure was defined as not being able to complete a full repetition without assistance or failing to keep up the standardized tempo set by the test leader on two consecutive repetitions. Specifically, if a participant included too long of a pause (> 1 s) between muscle actions, a warning from the test leader was provided; if the same occurred in the next repetition, this was deemed as muscular failure, thus denoting the end of the test. Within 15 s of completion of the test, the participants were required to rate their perceived pain on an 11-point numerical rating scale, with the instructions that 0 points were equivalent to “no pain” and 10 points were their “worst imaginable pain” [26] (Table 2).

**Table 2:** Mean ± standard deviation for each condition

Variable	Placebo	Caffeine	Caffeine and CitMal	CitMal
Jump height (cm)	31.1 ± 7.2	31.7 ± 7.4	31.8 ± 7.2	31.1 ± 8.0
Rate of force development (kN/s)	8.5 ± 3.7	8.6 ± 3.6	8.5 ± 3.9	8.1 ± 3.1
Peak force (N/kg)	20.8 ± 3.1	21.1 ± 3.4	21.2 ± 3.3	20.5 ± 3.2
Maximal power (W/kg)	14.3 ± 1.6	14.4 ± 1.7	14.4 ± 1.7	14.2 ± 1.9
1RM squat (kg)	119.9 ± 40.2	122.3 ± 41.5	124.3 ± 44.7	119.1 ± 41.3
Repetitions to failure in the squat	19.8 ± 4.8	22.7 ± 5.3	22.4 ± 6.0	21.9 ± 4.8
1RM bench press (kg)	84.2 ± 34.0	86.4 ± 35.4	85.9 ± 34.8	84.9 ± 35.3
Repetitions to failure in the bench press	19.2 ± 3.5	20.7 ± 3.3	21.4 ± 3.0	20.5 ± 4.0
Pain perception following the squat (0–10 scale)	5.4 ± 2.3	4.9 ± 2.3	5.0 ± 2.2	5.0 ± 2.2
Pain perception following the bench press (0–10 scale)	4.0 ± 2.1	4.0 ± 2.1	3.7 ± 2.0	3.8 ± 2.2

### Statistical analyses

The normality of data distribution was examined and confirmed using the Shapiro–Wilk test. A one-way analysis of variance (ANOVA) with repeated measures was conducted to explore the differences in performance outcomes (jump height, RFD, force, power, 1RM squat, 1RM bench press, number of repetitions in the squat and bench press) and subjective responses (pain perception) between the four conditions. In the case of a significant main effect from the ANOVA, the Sidak post hoc test was used to identify where the difference occurred (i.e., between which pairs). If the assumption of sphericity was violated, the Greenhouse–Geisser adjustment was used. All performance results are presented as mean ± standard deviations. Effect size (*d*) was calculated for pairwise comparison according to Cohen [27]. The magnitude of *d* was classified under the following thresholds, trivial (< 0.20), small (0.20–0.49), moderate (0.50–0.79), and large (≥ 0.80). In addition, mean differences between the conditions and their 95% confidence intervals were also calculated. The statistical significance threshold was set at *p* < 0.05. All statistical analyses were performed in SPSS version 27.0 (IBM Corp. Armonk, New York, USA).

## Results

### Countermovement jump

There was no significant main effect for CMJ height [F(3, 31) = 1.28; *p* > 0.05], RFD [F(3, 31) = 1.11; *p* > 0.05], peak force [F(3, 31) = 1.73;* p* > 0.05], or peak power [F(3, 31) = 0.59; *p* > 0.05]. As there was no significant main effect, no post hoc analyses were performed.

### Muscular strength

**Fig. 3:**
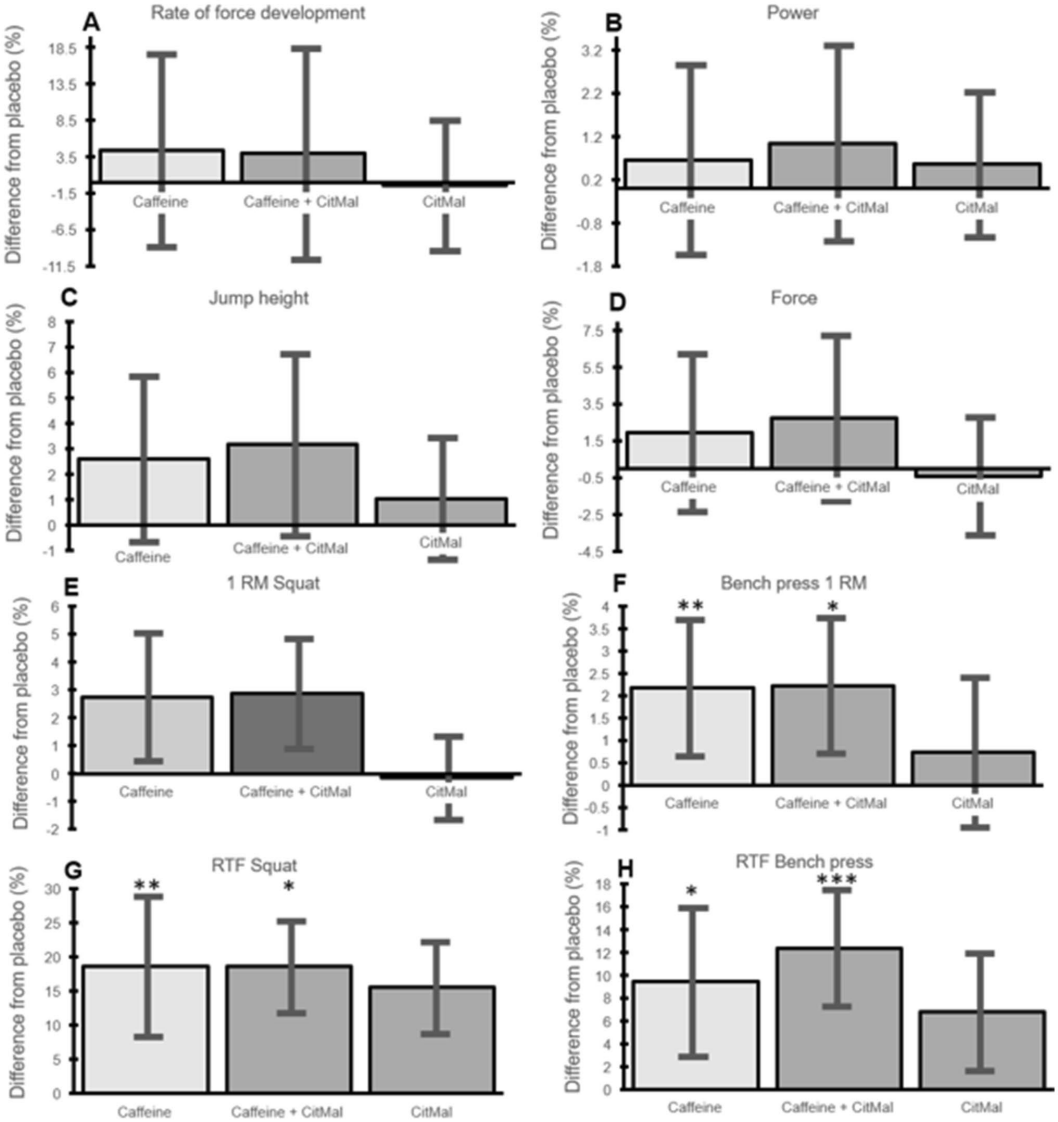
Percentage difference compared to placebo. Mean ± 95% confidence interval for **A** Rate of force development, **B** Power, **C** Jump height, **D**, Force, **E** 1 RM Squat, **F** 1 RM Bench press, **G** RTF Squat, and **H**, RTF Bench Press. 1 RM 1 repetition maximum, RTF Repetitions to failure. *Indicates significant different from placebo (p < 0.05), **Indicates significant differences from placebo (p < 0.01), and ***Indicates significant differences from placebo (p < 0.001)

**Fig. 4:**
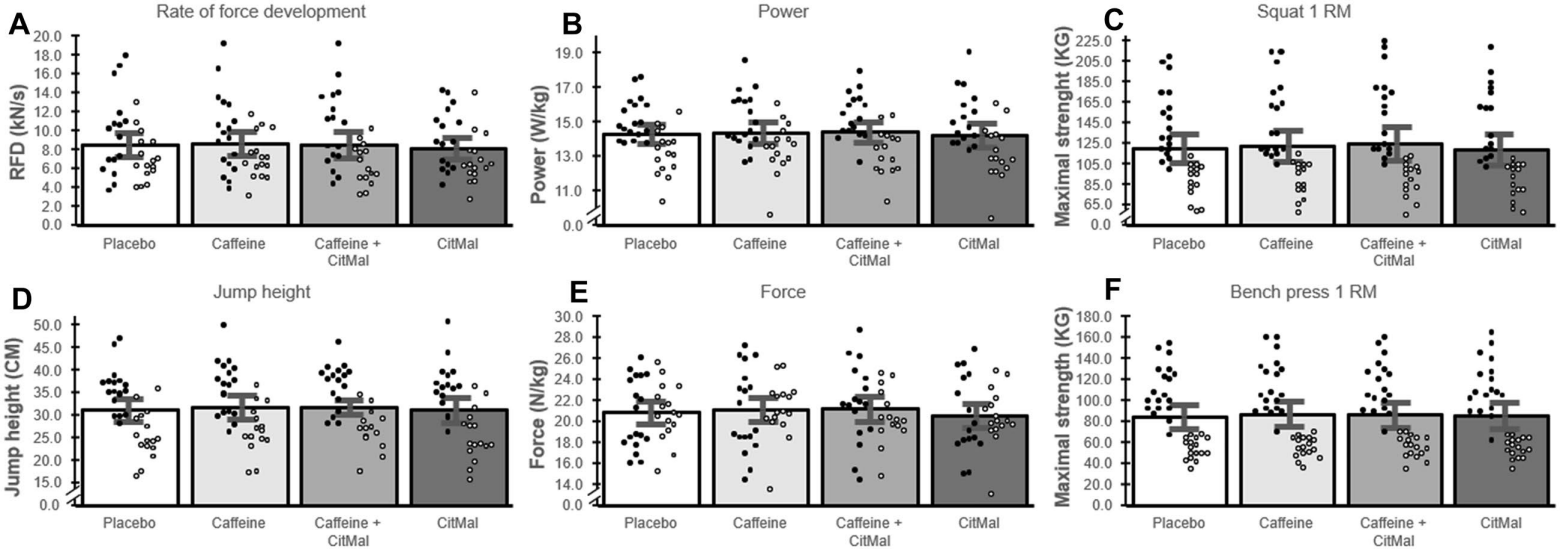
Data are presented as group mean ± 95% confidence interval and individual data (dark points are males and white points are females) for **A** Rate of force development, **B** Power, **C** Squat 1RM, **D** Jump height, **E** Force, and **F** Bench press 1RM. 1RM 1 repetition maximum

**Fig. 5:**
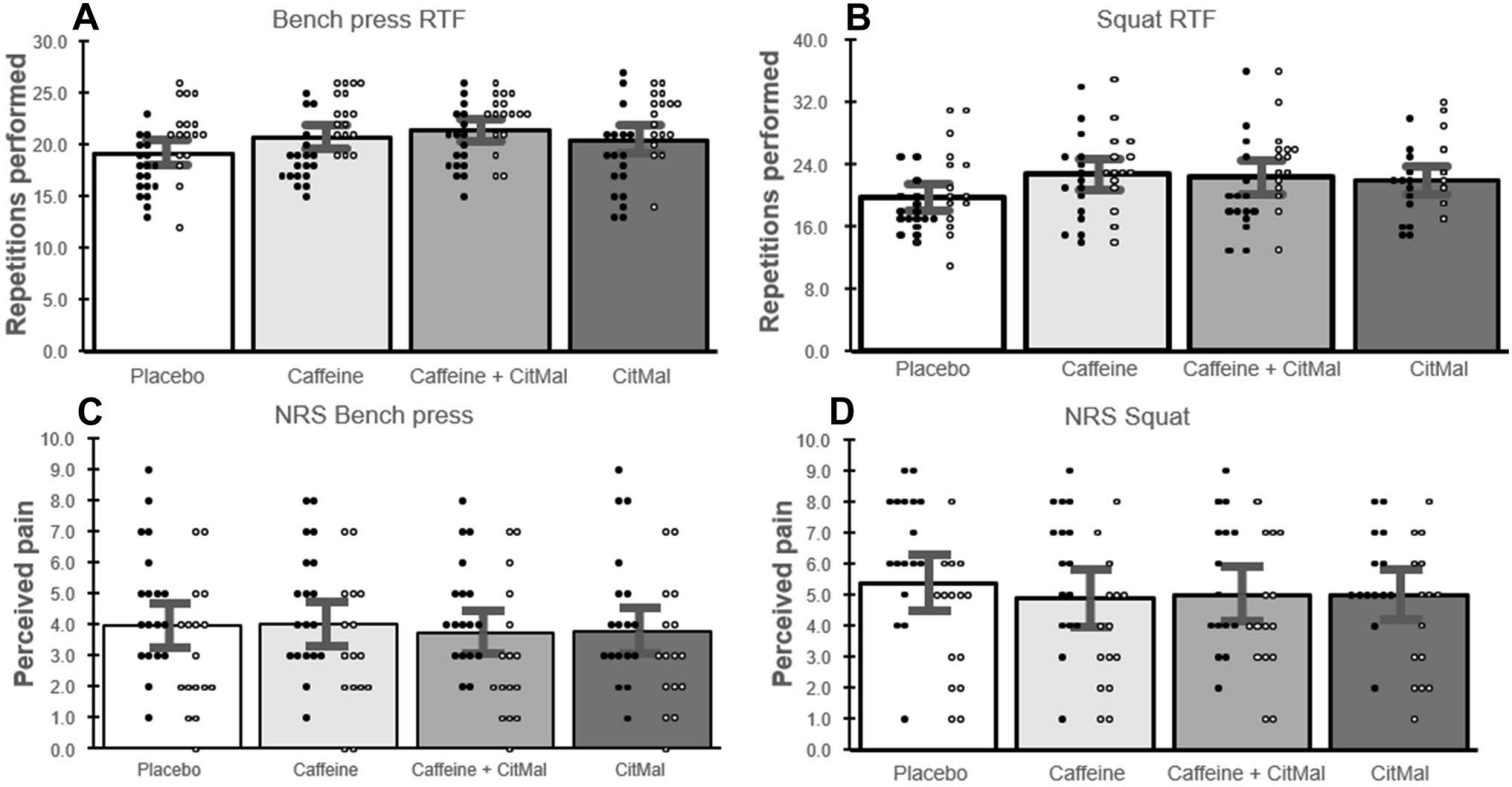
Data are presented as group mean ± 95% confidence interval and individual data (dark points are males and white points are females) for **A** Bench press repetitions to failure, **B** Squat repetitions to failure, **C** Numerical rating scale (pain) bench press, and **D** Numerical rating scale (pain) for squat. NRS numerical rating scale, RTF repetitions to failure

Percent change in strength from placebo to caffeine and CitMal supplementation are presented in Fig. 3, while Figs. 4 and 5 show absolute strength data with each condition. A significant main effect was found in the 1RM bench press [F(3, 33) = 4.56, *p* = 0.005]. Compared to the placebo condition, post hoc analyses revealed that isolated caffeine ingestion (Cohen’s *d*: 0.06; 2.7%; *p* = 0.004) and the combined ingestion of caffeine and CitMal enhanced 1RM bench press strength (Cohen’s *d*: 0.05; 2.5%; *p* = 0.022). There was no significant main effect for 1RM squat [F(3, 29) = 1.95, *p* = 0.17], and no other significant differences in any of the other pairwise comparisons (Table 3).

**Table 3:** Summary of the pair wise comparisons between the conditions

Outcome	Comparison	Mean difference (95% confidence interval)
1RM bench press	Placebo vs. caffeine	2.3 kg (0.6, 4.0)
	Placebo vs. CitMal	0.9 kg (− 1.2, 3.0)
	Placebo vs. caffeine and CitMal	1.9 kg (0.2, 3.6)
	Caffeine vs. CitMal	− 1.5 kg (− 3.8, 0.9)
	Caffeine vs. caffeine and CitMal	− 0.4 kg (− 1.8, 1.0)
	CitMal vs. caffeine and CitMal	1.0 kg (− 1.1, 3.1)
Repetitions to failure in the squat	Placebo vs. caffeine	3.0 repetitions (0.9, 5.1)
	Placebo vs. CitMal	1.9 repetitions (− 0.3, 4.0)
	Placebo vs. caffeine and CitMal	2.5 repetitions (0.2, 4.9)
	Caffeine vs. CitMal	− 1.2 repetitions (− 3.4, 1.1)
	Caffeine vs. caffeine and CitMal	− 0.5 repetitions (− 2.8, 1.6)
	CitMal vs. caffeine and CitMal	0.6 repetitions (− 2.1, 3.4)
Repetitions to failure in the bench press	Placebo vs. caffeine	1.6 repetitions (0.3, 2.9)
	Placebo vs. CitMal	1.2 repetitions (− 0.04, 2.4)
	Placebo vs. caffeine and CitMal	2.1 repetitions (0.9, 3.3)
	Caffeine vs. CitMal	− 0.4 repetitions (− 1.9, 1.1)
	Caffeine vs. caffeine and CitMal	0.5 repetitions (− 0.5, 1.6)
	CitMal vs. caffeine and CitMal	0.9 repetitions (− 0.4, 2.2)

### Muscular endurance

A significant main effect was found for the number of repetitions in the squat [F(3, 29) = 5.40, *p* < 0.001]. As compared to the placebo condition, post hoc analyses revealed an increase in the number of performed repetitions following isolated caffeine ingestion (Cohen’s *d*: 0.58; 18.7%; *p* = 0.002) and combined caffeine and CitMal ingestion (Cohen’s *d*: 0.46; 18.6%; *p* = 0.025). There were no other significant differences in any of the other pairwise comparisons.

A significant main effect was found for the number of repetitions in the bench press [F(3, 33) = 7.66, *p* < 0.001]. As compared to the placebo condition, post hoc analyses revealed an increase in the number of performed repetitions following isolated caffeine ingestion (Cohen’s *d*: 0.48; 9.5%; *p* = 0.012) and combined caffeine and CitMal ingestion (Cohen’s *d*: 0.64; 9.3%; *p* < 0.001). There were no significant differences in any of the other pairwise comparisons.

There was no significant effect for pain perception following the completion of the repetitions to failure test in squat [F(3, 29) = 0.73, *p* = 0.54] and bench press [F(3, 33) = 0.96, *p* = 0.42].

### Effectiveness of the blinding

In the effectiveness of the blinding evaluation, 46% (16/35), 46% (16/35), 26% (9/35), and 23% (8/35) of the participants correctly guessed the placebo, caffeine, caffeine and CitMal, and isolated CitMal conditions, respectively.

### Adverse events

Seven adverse events were reported. These adverse events included headache (three), nausea (three), and dizziness (one). Six out of seven adverse events were reported as ‘probably’ when investigating the relationship to the supplements, and one adverse event was categorized as ‘definite’. Six adverse events that were categorized as ‘probably’ were also considered ‘non-serious’. From an intensity standpoint, three adverse events were categorized as ‘mild’, and four as ‘moderate’. Notably, all seven adverse events were reported when participants received both caffeine and CitMal combined, and five out of the seven adverse events were reported by female participants. Finally, besides these seven events, one participant threw up 22 min after supplement ingestion of both caffeine and CitMal. For this participant, testing that day was terminated and successfully conducted another day.

## Discussion

This is the first study to explore the effects of co-ingestion of caffeine (5 mg/kg) and CitMal (12 g) compared to either supplement in isolation or placebo. The main findings of this study were that the isolated ingestion of caffeine and co-ingestion of caffeine and CitMal was ergogenic for upper-body muscular strength and endurance, and lower-body muscular endurance. We did not detect any additional effects of co-ingesting CitMal with caffeine, suggesting that combining these supplements may not provide additive effects compared to isolated caffeine ingestion. For CMJ variables, and pain perception, there were no differences between the treatments, and CitMal supplementation in isolation did not have an ergogenic effect on any outcome. As such, these results only partially confirm our initial hypothesis.

### Countermovement jump

Caffeine consumption alone and combined with CitMal did not influence jump height in our study. The lack of an effect on CMJ measurements is contrary to other research on caffeine [7, 28]. Specifically, previous meta-analyses on the effects of caffeine on jump height reported an ergogenic effect in athletes [29–31] and non-athletes [32]. While we did not find an ergogenic effect of caffeine on jump height, it is relevant to mention that the effect size favored the two conditions where caffeine was provided (*d*: 0.08–0.10). While the effect size was small, it should be mentioned that small effect sizes are observed in previous meta-analytical data (Hedges’ *g*: 0.17; Grgic et al. 2018). Thus, it might be that the lack of an effect in the present study is due to the variability in responses to caffeine between individuals [33]. Besides jump height, we also did not find a significant effect on other CMJ-derived outcomes, such as RFD, peak force, and peak power. Caffeine has been shown to elicit a significant effect on RFD when tested during resistance exercises, but not during vertical jumps, which could explain the lack of improvements in our study [28].

Besides the two treatments with caffeine, isolated ingestion of CitMal did not enhance jumping performance. Rapid force production is necessary to enhance performance in the CMJ test, which is not likely to be achieved with CitMal ingestion. Indeed, CitMal ingestion is likely to be ergogenic in exercise of longer duration, whereas the performance benefit during short burst exercises is likely to be trivial or non-existential. Our results for jumping performance are similar to those from Glenn et al. [34], who reported that peak or average vertical jump power was not enhanced following the ingestion of 8 g of CitMal.

### Muscular strength

In the present study, caffeine alone and caffeine plus CitMal increased maximal strength compared to placebo. The ergogenic effects of caffeine on maximal strength are well-established [3, 4, 7]. Contemporary meta-analyses have demonstrated an ergogenic effect of caffeine ranging from 0.16 to 0.20 [2–4, 7]. In the present study, the effect sizes were very small (*d*: 0.05–0.06; 2.5–2.7%; mean difference of ∼2 kg). It seems likely that these differences could be valuable only for athletes in strength-based sports like powerlifting. While we did not include powerlifters as participants, it should be mentioned that a few participants in our study lifted above 200 kg in the squat and above 140 kg in the bench press (Fig. 4). None of the lifters were competitive powerlifters, but such numbers are similar to previous observations in some national-level powerlifters [35]. Our findings largely mirror those from Grgic et al. [36], who provided participants with 2, 4 and 6 mg/kg and found that only the two higher doses enhanced upper-body strength by an effect size of *d* = 0.07–0.09. Overall, caffeine ingestion—either in isolation or in combination with CitMal—may enhance 1RM strength by a small magnitude.

While caffeine provided an ergogenic effect, isolated CitMal ingestion did not influence maximal strength. In the currently available studies exploring CitMal, there is a distinct lack of those that evaluated the effects of CitMal on 1RM performance. However, they have used other measurements of maximum strength. For example, one study provided 8 g of CitMal and evaluated peak force during different muscle actions. In line with our 1RM data, the ingestion of CitMal did not enhance isometric, concentric, or eccentric peak force [37]. In addition, a recently published meta-analysis [38] also did not find an effect of CitMal supplementation on muscle strength. However, caution should be taken when interpreting this meta-analysis, given that only four studies were included. In summary, as the present study is the first to explore CitMal effect on 1RM strength, more research in the field is needed.

### Muscular endurance

Caffeine alone and caffeine co-ingested with CitMal improved lower-body and upper-body muscular endurance with a moderate effect size. While an ergogenic effect was found in both conditions, there were no differences between isolated caffeine vs. caffeine and CitMal combined.

An ergogenic effect of caffeine on muscular endurance has been commonly observed in the literature. For example, Norum et al. [39] provided strength trained female participants with 4 mg/kg of caffeine and observed an ergogenic effect on muscular endurance in the squat and bench press; results that echo ours. Filip-Stachnik et al. [40] used 6 mg/kg and also reported that caffeine ingestion enhanced upper-body muscular endurance in the bench press exercise in female participants. In addition to the primary studies, several meta-analyses have also established an ergogenic effect of caffeine on muscular endurance while pooling data from: (a) isometric, isokinetic, and isotonic tests [41]; (b) only isotonic tests [42]; or (c) using data only collected in females [43]. Overall, our results support and add to the body of evidence supporting an ergogenic effect of caffeine on muscular endurance in both men and women. These improvements are likely mediated by increased motor unit recruitment commonly observed following caffeine ingestion [44].

We did not detect a significant effect of isolated CitMal ingestion on muscular endurance. Still, it should be mentioned that the mean difference highly favored the CitMal condition compared to placebo. Mean differences favored CitMal in the squat by 1.9 repetitions (95% confidence interval: − 0.3, 4.0 repetitions) and in the bench press by 1.2 repetitions (95% confidence interval: − 0.04, 2.4 repetitions). In line with our findings, a recent meta-analysis by Vårvik et al. [17] indicated that CitMal has a small ergogenic effect and can enhance repetitions to failure by approximately 3 additional repetitions, somewhat lower than the mean difference observed herein. Thus, if there is an ergogenic effect on muscle endurance following CitMal ingestion, the effect is likely minor.

The results in the present study suggest that the combination of caffeine and CitMal is not likely to have additive ergogenic effects on muscular endurance. There are several possible reasons for such a lacking additive effect. First, when compared to placebo, isolated CitMal was not found to be ergogenic—even though the effects highly favoured the CitMal condition. Given that CitMal did not demonstrate significant ergogenic effects in isolation, it seems logical that adding this supplement to caffeine would not yield greater performance benefits than ingesting caffeine alone in our study. Nevertheless, since the potential ergogenic effect of CitMal on muscular endurance appears to be very small [17], it is possible that a type II error occurred and the present study was underpowered to detect such small differences, or that the effect is negligible. From an alternative perspective, it might also be that these supplements counteract each other. As mentioned previously, CitMal may increase vasodilatation, while caffeine may induce vasoconstriction, thus negating the potential ergogenic effect of CitMal and hence, reducing the probability of their synergistic effect [45]. More research is needed to elucidate the possible interactions from combined supplementation of caffeine and CitMal.

### Pain perception

In the analysis for pain perception, we did not find a significant main effect. Previous studies observed that caffeine ingestion reduces pain perception [46, 47], even though these findings are equivocal [48, 49]. It should be mentioned that the possible reduction in pain perception following caffeine ingestion may not determine caffeine’s ergogenic potential. Specifically, previous studies reported that improved exercise performance following caffeine ingestion can occur without any alterations in pain perception [48, 49]. Thus, our results add to the body of evidence supporting the notion that the ergogenic effects of caffeine on resistance exercise can be mediated by factors such as increased motor unit recruitment, not necessarily reduced pain perception.

### Adverse events

From a safety perspective, the co-ingestion of caffeine and CitMal could lead to gastrointestinal problems. Indeed, several participants reported nausea, but only following the combined ingestion of caffeine and CitMal. Interestingly, the incidence of side-effects was more common in women. The reason for that may be because the relative dose of CitMal was larger for women than men (0.14 g/kg for males and 0.18 g/kg for females). In addition, ingestion of caffeine alone has also been previously reported to cause gastrointestinal problems [50]. Thus, from a safety perspective, co-ingestion of these supplements may increase the incidence of adverse events.

### Limitations and strengths of the study

One of the limitations of the present study is that we did not measure plasma concentrations of caffeine and citrulline. Secondly, we did not measure possible mediators of the supplements that could have given more insight into the potential interactions between them, such as vasoconstriction and vasodilatation. Thirdly, the majority of participants in the present study were classified as moderate caffeine consumers (*n* = 11; between 1 and 3 mg/kg/day), and some were classified as low (*n* = 9; < 1 mg/kg/day) and high (*n* = 15; > 3 mg/kg/day) caffeine consumers [51]. It is possible that habitual intake of caffeine influence the ergogenic effects [52], but the literature is somewhat ambiguous on this topic [52].

Despite the outlined limitations, there are also certain strengths of the study that should be considered. For example, we included a fairly large number of both male and female participants. We evaluated multiple performance outcomes, including jumping-related outcomes (i.e., jump height, RFD, peak force, and peak power), muscular strength and endurance. The barbell back squat and bench press exercises were used to evaluate muscular strength and endurance, miming real-life conditions given the common use of these exercises in various strength and conditioning programs [21]. Finally, we provided data exploring the effects of combining caffeine and CitMal, which is also practically important given the rise in popularity of multi-ingredient pre-workout supplements [53].

## Conclusions

This is the first study to explore the effects of co-ingestion of caffeine (5 mg/kg) and CitMal (12 g) compared to either supplement in isolation or placebo. We found that the ingestion of caffeine alone or combined with CitMal improved maximal strength and muscular endurance, but there were no additive effects of combining these supplements. There was no significant difference between the conditions for CMJ-derived variables. CitMal provided in isolation was not ergogenic for any of the analyzed outcomes. In summary, caffeine ingestion appears to be ergogenic for muscular strength and muscular endurance, while adding CitMal does not seem to further enhance these effects.

## Supplementary Information

Below is the link to the electronic supplementary material.

Supplementary file1 (DOCX 153 KB)

## Data Availability

On reasonable request data can be shared.
