# Supplementary material for: Effect of isolated and combined ingestion of caffeine and citrulline malate on resistance exercise and jumping performance: a randomized double-blind placebo-controlled crossover study
Source: Eur J Nutr. 2023 Jul 14;62(7):2963–75. doi: 10.1007/s00394-023-03212-x (PMC10468939; doi:10.1007/s00394-023-03212-x)
Supplement: Supplementary file 1 — Supplementary file1 (DOCX 153 KB) [file 394_2023_3212_MOESM1_ESM.docx]

**Supplementary figure**


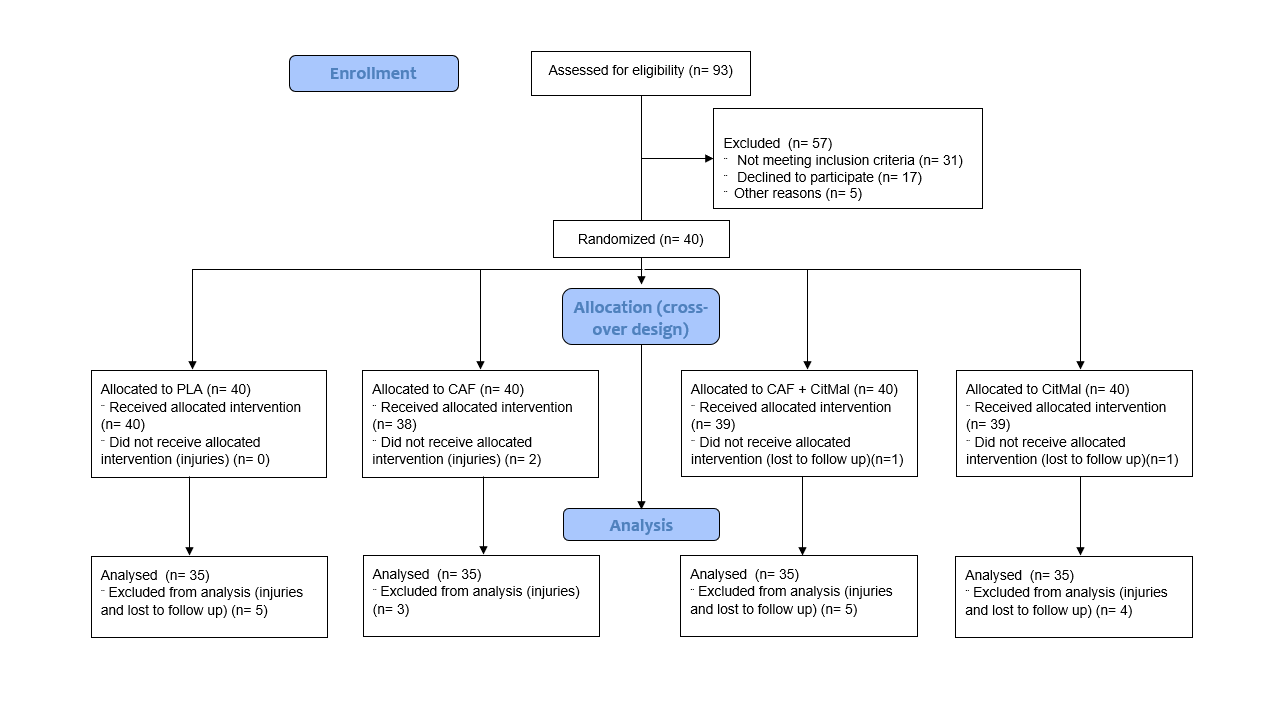


**Supplementary figure 1** Consort Flow Diagram for this within-subject trial. Diagram shows participant flow through each stage of the trial (enrolment, allocation, and analysis).
